# Feasibility of Emotional Awareness and Expression Therapy Adapted for Headache Disorders at a Tertiary Headache Clinic

**DOI:** 10.1017/cjn.2026.10573

**Published:** 2026-02-26

**Authors:** Orit Zamir, Brandon C. Yarns, Manav V. Vyas, Ana-Marissa Lagman-Bartolome, Ian Stanaitis, Ashna Jalan, Lina Jobanputra, Mary Jane Esplen, Mateusz Zurowski, Valerie Lawler, Christine Lay

**Affiliations:** 1 Department of Psychiatry, University of Toronto, Toronto, Ontario, Canada; 2 Women’s College Hospital, Toronto, Ontario, Canada; 3 University of California Los Angeles David Geffen School of Medicine, Los Angeles, CA, USA; 4 Division of Neurology, Department of Medicine, University of Toronto, Toronto, Ontario, Canada; 5 Department of Pediatrics, Division of Neurology, Children’s Hospital, London Health Sciences Center, Schulich School of Medicine & Dentistry, University of Western Ontario, London, Ontario, Canada

**Keywords:** Adverse childhood experiences, emotional awareness, headache, migraine, psychological treatment

## Abstract

**Objective:**

Chronic headache, including migraine, is often associated with psychiatric conditions and adverse childhood experiences. This study examines the feasibility of Creating Calm (CC), a modified form of Emotional Awareness and Expression Therapy (EAET) that targets the emotional impacts of adversity in headache patients at a tertiary headache center. We also explored changes in headache days, disability, psychosocial well-being and possible mechanisms to plan for a randomized controlled trial.

**Methods:**

We conducted a prospective single-arm pragmatic pilot study to evaluate the feasibility of CC delivered as nine weekly group telehealth sessions in a tertiary headache clinic for adults with high-frequency episodic and chronic headache. Continuation of medical treatments was consistent with the pragmatic design. CC integrates education, mindfulness, cognitive and behavioral approaches, with an emotion-focused mind-body approach used in EAET. Feasibility was based on recruitment, retention and adherence measures. Acceptability was measured through participant satisfaction. We also explored changes in headache, psychosocial and mechanism measures before, after, and 2 months post-treatment.

**Results:**

Of the 33 participants recruited, 30 (91%) completed at least 7 out of 9 sessions, and 28 (85%) completed surveys. Participants reported satisfaction with the intervention (mean [SD] 47.6 [10.4] out of 60 Satisfaction with Therapy and Therapist Scale-Revised [STTS-R]). Exploratory analyses found a signal of reduction in headache days per month (mean [SD], 20.8 [7.6] to 15.5 [7.8], *p* = 0.004), disability, depression and improvement in global mental health following intervention.

**Conclusion:**

This real-world pilot study supports the feasibility and acceptability of modified EAET for patients with headache, warranting a prospective randomized clinical trial.

## RÉSUMÉ

La faisabilité de la thérapie de la prise de conscience et de l’expression des émotions, adaptée aux troubles céphalalgiques, dans un centre de soins tertiaires de traitement des céphalées.

**Objectif:**

Les céphalées chroniques, dont la migraine, sont souvent associées à des troubles psychiatriques et à des expériences difficiles vécues durant l’enfance. L’étude ici décrite visait à examiner la faisabilité de la mise sur pied d’une intervention de recherche du calme (RC), une version modifiée de la thérapie de la prise de conscience et de l’expression des émotions (TCEE), qui cible les effets émotionnels de l’adversité chez les personnes souffrant de troubles céphalalgiques, dans un centre de soins tertiaires de traitement des céphalées. L’équipe de recherche s’est également penchée sur les changements du nombre de jours de céphalée, d’incapacité, de bien-être psychosocial et de mécanismes possibles d’évolution en vue de la planification d’un essai comparatif, à répartition aléatoire.

**Méthode:**

Il s’agit d’une étude pilote, pragmatique, prospective, à cohorte unique, visant à évaluer la faisabilité de la mise sur pied d’un programme de RC, organisé en neuf séances hebdomadaires de télésanté en groupe, dans un centre de soins tertiaires de traitement des céphalées, pour les adultes connaissant des céphalées chroniques ou des épisodes très fréquents de céphalée. La poursuite du traitement médical faisait partie du cadre pragmatique de l’étude. La thérapie par la RC intègre à la fois la formation, la pleine conscience et les approches cognitive et comportementale, de même que l’approche de l’esprit et du corps axée sur les émotions, appliquée dans la TCEE. La faisabilité reposait sur le recrutement et la rétention des sujets ainsi que sur les mesures de respect du protocole. L’acceptabilité se mesurait par la satisfaction des participants et des participantes. Enfin, il y a eu des mesures de changements de céphalées, de bien-être psychosocial et de mécanismes d’évolution avant le traitement, après celui-ci et 2 mois plus tard.

**Résultats:**

Sur 33 participants et participantes à l’étude, 30 (91 %) ont assisté à au moins 7 séances sur 9, et 28 (85 %) ont répondu aux différents questionnaires. Les sujets ont fait état de leur degré de satisfaction à l’égard de l’intervention (moyenne [écart type : σ] : 47,6 (10,4) sur 60 [Échelle de Satisfaction avec la Thérapie et le/la Thérapeute – Version Révisée (STTS-R)]. Enfin, une tendance à la réduction du nombre de jours par mois de céphalée (moyenne [σ] : 20,8 [7,6] à 15,5 [7,8]; *p* = 0,004), d’incapacité et de dépression ainsi qu’à une amélioration de la santé mentale générale s’est dégagée des analyses préliminaires effectuées à la suite de l’intervention.

**Conclusion:**

Cette étude pilote en monde réel confirme la faisabilité et l’acceptabilité de la mise en œuvre d’une version modifiée de la TCEE pour les personnes souffrant de céphalées, constatation justifiant la réalisation d’un essai clinique prospectif, à répartition aléatoire.

Highlights

- Chronic headache conditions are hard to treat, partly due to high rates of adversity and comorbidities.
- This open-label real-world pilot study evaluated *modified Emotional Awareness and Expression Therapy (EAET)*, a psychological group treatment focusing on emotional processing of adversity.
- Modified EAET is feasible and acceptable and should be further studied.

## Introduction

Headache disorders are the third leading global cause of disability,^1^ and migraine is the second leading cause of disability for disorders affecting the nervous system in adults.^2^ Risk factors for migraine chronicity include psychiatric comorbidities and adverse childhood experiences (ACEs).^3^ When these comorbidities are present, the American Headache Society Consensus statement (2021) recommends non-pharmacological treatments in addition to medication, including behavioral interventions.^4^ A recent meta-analysis of behavioral interventions, which included cognitive behavioral therapy (CBT) and mindfulness-based interventions (MBIs), concluded that there may be a reduction in migraine frequency of approximately 1–2 headache days per month compared to a mix of control groups (active and waitlist), as well as reduced disability in a population with a median baseline headache frequency of 10 per month.^5^ Another recent Cochrane review showed mixed results.^6^ Optimizing treatment may include exploring alternative potential psychosocial drivers of headaches that CBT and MBIs may not directly address. Since psychiatric comorbidities and childhood adversity are common and predict a worse prognosis, a psychological approach focusing on emotional processing of adverse experiences may improve outcomes, especially in tertiary care settings, where patients frequently have high baseline comorbidity, migraine disability and chronicity.^7^

One treatment approach that addresses the consequences of developmental adversity and trauma (i.e., ACEs) is Emotional Awareness and Expression Therapy (EAET).^8^ This novel manualized psychological treatment, delivered in group, individual or asynchronous telehealth formats, demonstrates medium to large effect size benefits in those living with chronic pain^9–12^ and somatic symptom disorders.^13,14^ As an experiential mind-body therapy, EAET utilizes a psychodynamic and emotion-focused framework along with pain neuroscience education. EAET encourages adaptive experiencing, expression and releasing of avoided challenging emotions to reduce chronic pain symptoms.^15^ Key EAET techniques are derived from Intensive Short-Term Dynamic Psychotherapy (ISTDP), a lengthy and complex format of individual psychotherapy with an evidence base for various somatic symptom presentations,^16^ including headache,^17^ which may be difficult to scale up due to the extensive training required for mastery of this modality. Although several EAET studies included some types of headache conditions,^9,10,12^ no prior study has specifically evaluated the effects of EAET in patients with chronic headache in tertiary care settings, who often have high levels of anxiety and affect dysregulation, as well as significant mental health comorbidities and ACEs.^7^ For this complex population, the most suitable and scalable treatment may be a modified format of EAET integrated with widely known and evidence-based cognitive-behavioral and mindfulness techniques for headache – a treatment we called *Creating Calm* (*CC*).

In this pragmatic real-world study, we evaluated the feasibility and acceptability of delivering *CC* for the complex patients who present to a tertiary care clinic with high-frequency episodic and chronic headache. We hypothesized that this intervention would be feasible and acceptable.

## Methods

### Study design and procedures

We conducted a single-arm open-label prospective exploratory pilot feasibility study from April 2023 to May 2024 to evaluate the feasibility and acceptability of *CC*, a psychological telehealth group intervention, in a tertiary headache center. The study was approved by the Women’s College Hospital (WCH) Research Ethics Board (REB# 2022-0150-E). The study was explained over the phone, and participants had an opportunity to ask questions and think about responses before providing electronic informed consent. Informed consent and all other research forms and questionnaires were sent to participants and completed through the secure electronic platform REDCap. Renumeration ($25 gift certificate) was provided following completion of each set of questionnaires at baseline, post-treatment and 2-month follow-up period.

### Participants

Participants were recruited from the Centre for Headache, a tertiary outpatient clinic in Toronto, Ontario, Canada. The Centre for Headache population is complex and treatment refractory, having failed primary and secondary headache care and, often, several trials of specialized tertiary headache pharmacological and interventional treatments. In recruiting from this population, the study had an explicit focus on improving equity for complex patients who are excluded from most studies, making it a pragmatic, real-world approach. We included patients who were 18 years old and older and had a history of primary headache, including migraine, with a frequency of 8 or more days per month (i.e., high-frequency episodic or chronic headache). Headache diagnosis and frequency were determined by a headache neurologist or nurse practitioner using the International Classification of Headache Disorders-3 criteria. Exclusions were few and included those unable to consent, unable to speak or read English or without internet access and those with a secondary headache disorder as their principal headache diagnosis. In addition, for safety purposes, individuals with severe and unstable psychiatric conditions such as schizophrenia, untreated bipolar disorder, active suicidal ideation or active substance use disorder were excluded. Also consistent with pragmatic design,^18^ participants continued their usual care throughout the study; however, they were restricted from taking part in another group psychological intervention.

### Intervention

Following consent, two group facilitators invited participants to a 20-minute video telehealth intake session to review the therapy model, build a therapeutic alliance and prepare participants for the group. *CC* was a 9-week manualized psychological telehealth group intervention for headache that integrated EAET with elements of anxiety regulation, cognitive restructuring, relaxation training, mindfulness and headache education. A group-based intervention was chosen, as it takes less therapist time compared to individual therapy, leading to a more sustainable and scalable treatment option beyond the end of the trial, and because group-based EAET has been shown to be robustly effective for chronic musculoskeletal pain.^9^ Telehealth and group format also served an equity goal, allowing increased access to the study, as this tertiary care clinic serves a large regional population. All modalities integrated into *CC* have all previously been conducted in group settings. This program was developed by a multidisciplinary team consisting of a psychiatrist (O.Z.) and social worker (L.J.) at the Centre for Headache, with consultation from local and international experts in the field of headache and pain medicine (C.L., V.L., AM.L.B., B.C.Y., M.Z.) and anonymous feedback from patients following group completion before and during the study. The development of program content and participant material included a literature review of key information regarding migraine behavioral and psychological interventions, summarized in Zamir *et al*. (2023).^7^ O.Z. developed detailed facilitator and participant manuals for *CC*, with support from L.J. and B.C.Y., which included psychoeducation, worksheets and experiential exercises based on CBT, MBIs, trauma-focused anxiety regulation and EAET.^19^ Sessions were 105 minutes in duration for the first cycle and lengthened to 120 minutes for the following two cycles, based on participant feedback during this exploratory pilot study, and included approximately 10–12 participants per group. Sessions used WCH’s security-protected Zoom virtual video format within the EPIC electronic medical record system. The two group facilitators were experienced mental health clinicians in the field of medical care.

**Figure 1.**
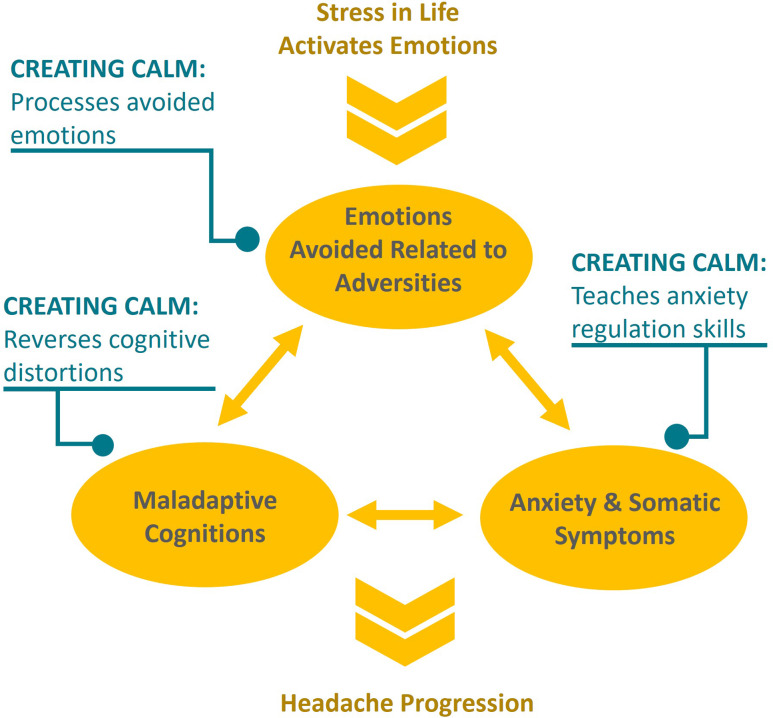
Creating Calm targets headache progression. *Note:* Creating Calm targets three components proposed to be responsible for headache progression by encouraging (1) emotional processing to address avoided emotions associated with life stressors, (2) identification of defenses and cognitive restructuring to target maladaptive cognitions and defenses and (3) identification and regulation skills to target anxiety and somatic symptoms.

*CC* targeted three components of headache progression (Figure 1), which included (1) anxiety regulation, (2) identifying emotional avoidance and cognitive restructuring and (3) emotional processing. Table 1 summarizes each session’s content. Briefly, mindfulness and relaxation practices started and ended all sessions to support awareness, regulation and processing experiences. Session *one* included neuroscience education based on the EAET model and tailored for headache patients (i.e., headache progression can occur with the avoidance of stressful emotional experiences, thereby changing brain circuitry). Session *two* included a multicomponent approach to anxiety and its optimization to promote better functioning. Two models of anxiety were integrated. Participants were introduced to the ISTDP model of three anxiety “channels”: striated muscle anxiety (e.g., muscle tension in the back or neck), smooth muscle anxiety (e.g., tension in the smooth muscle of the gut or blood vessels leading to so much anxiety that one develops nausea, vomits or has a headache) and mental processes (e.g., being so anxious that one cannot think clearly).^20^ Identifying the channel can help participants identify and label anxiety manifestations in the body so that they may be regulated into what is viewed as the “healthier” striated muscle channel. The three anxiety channels were mapped onto and integrated with a second trauma-based model, the window of tolerance,^21^ which focuses on maintaining anxiety at an “optimal,” or moderate, level to promote better functioning. The session also presented several anxiety regulation strategies, such as grounding. Session *three* introduced the concept of emotional avoidance based on maladaptive, distorted cognitions and the psychodynamic concept of defenses. Starting session *four*, emotional processing was the primary focus. The goal was to process current life experiences and link them to early adverse events through experiential practices. Each week, education was provided on one of the adaptive emotions outlined in the EAET/ISTDP framework, including anger, guilt, grief and love, which are commonly in conflict and avoided in relationships due to life adversities.^19^

**Table 1.** *Creating Calm* session content

Week	Theme	Content*
1	Introduction	Model of *Creating Calm* introduced using neuroscience education and the triangle of headache progression (Figure 1). This model proposes three components responsible for headache progression: (1) avoided emotions related to adversity and stress, (2) maladaptive cognitions and defenses and (3) anxiety and symptom manifestation. Program task was introduced: identify (1) and (2) and regulate (3). Brief review of headache lifestyle education, including common triggers.
2	Anxiety regulation	Education for somatic symptom manifestation of anxiety using two models: The different ways anxiety can be felt in the body based on psychodynamic understanding.^19^ Window of tolerance: Trauma-informed model of arousal. Review and practice of anxiety regulation based on grounding practices and STOPP (stop, take a breath, observe anxiety and thoughts, pull back from unhelpful strategies, proceed to process underlying stressor and emotional content). Brief introduction to sleep hygiene tips.
3	Emotional avoidance	Introduction to common defenses (i.e., strategies to avoid unacceptable internal experiences, such as feelings) based on psychodynamic therapy, cognitive distortions (e.g., catastrophizing) from cognitive models and internal family systems (e.g., inner critic part). Participants encouraged to separate from avoidance strategies through examining cost, mindfulness and parts work.
4	Emotional processing: *overview*	Introduction to labeling the function and properties of emotions (i.e., cognitive component “I am angry”; somatic component “I feel anger in my chest”; impulses “Anger make me want to lash out”). Experiential exercise to introduce the components of emotions using pride.
5	Emotional processing: *anger*	Introduction to the function and experience of the core emotion anger First practice of emotional experiencing, expressing and releasing (EER). Participant shares recent stressful event with the encouragement to experience and release emotion and other intense or difficult emotions using visualization/imagination.
6	Emotional processing: *guilt, shame*	Introduction to the concept and expression of healthy and unhealthy guilt and shame. Self-compassion skills. EER experiential exercise.
7	Emotional processing: *sadness, love*	Differentiating sadness, an expression of grief, from depression, a maladaptive avoidance of emotional experiencing. EER experiential exercise.
8	Communication	Introduce a spectrum of healthy communication and a framework for communicating emotions and challenging conversations. EER experiential exercise with a focus on communication.
9	Summary and next steps	Review of the program. Patients summarize benefits, insights and continued work structured into a “Wellness Planner.”

* Homework introduced for all sessions, with emphasis in sessions 1–3 on building a relaxation and grounding practice and sessions 4–8 on written exercises to encourage emotional experiencing.

A key part of EAET is to process stress and adversity-related emotions based on the notion that chronically avoiding emotions results in symptoms.^8,19^ Emotions faced directly in the safe environment of the therapy setting, through experiencing, expressing and releasing (EER) practices, support symptom reduction or elimination. EER, informed by psychodynamic psychotherapy, encourages participants to experience emotions in the body (e.g., anger with sensations of heat and energy), express the feeling in words and imagination and then release it (e.g., say goodbye to the anger, let it go).^8^ The emphasis is on guiding the participants toward emotions they have been avoiding, allowing them to experience empowerment and a new experiential understanding of events in which these emotions were initially avoided due to threatening or dismissive messages from caregivers or others. Participants who may be detached from emotions related to adversity and have significant anxiety can be easily overwhelmed, which is commonly seen with chronic migraine and headache.^3,22^ In such cases, a graded approach is used to process emotions, which includes anxiety regulation and integration strategies from ISTDP, CBT and MBI practices, as well as initially guiding participants toward life experiences where emotions can be more readily accessed (e.g., anger after getting cut off in traffic) before examining more traumatic experiences. Further processing is encouraged through written emotional expression exercises assigned for homework. In the later weeks, we also introduced adaptive relational communication strategies.

### Assessments

Once consented, participants completed baseline demographic, clinical history and outcome questionnaires. Participants also downloaded headache diaries to be completed manually for the month before initiating the intervention and 2 months following the intervention. Baseline demographics and clinical history included gender, education, race/ethnicity, marital status, employment, income, trauma history detemined using the Life Events Checklist for Diagnostic and Statistical Manual of Mental Disorders-Fifth Edition (DSM-5)^23^ and pain history. Chart review by a trained research assistant gathered data on sex, age, headache age of onset, diagnosis, frequency, medications, as well as psychiatric diagnoses and ACE history. As part of standard practice in the clinic, neurologists and nurse practitioners had systematically inquired about ACEs during the initial clinic consultation and recorded this information in the chart.

### Outcomes

Our primary outcome was the feasibility and acceptability of *CC*. The feasibility outcome was based on recruitment (i.e., number screened and enrolled in program) as well as adherence to protocol (i.e., survey completion) and retention (i.e., attendance). The aim for each group cycle was to recruit enough people for 10–12 participants in each group for a total of 30–36 participants in the study over the duration of 12–14 months. Optimal program completion was defined as 80% or more of participants attending 7 out of 9 sessions (i.e., 75% of the program sessions).^24^ We also reported the percentage of people who completed questionnaires. We evaluated acceptability as satisfaction with the program, which was measured using the Satisfaction with Therapy and Therapist Scale-Revised (STTS-R)^25^ scale, a validated tool to measure satisfaction with therapy. This was completed by the participants at the end of the intervention. The score includes 12 items for therapy and therapist satisfaction (range 12–60) and 1 item for global impression of change (range 1–5).

To support planning for a future randomized control trial, we also explored headache frequency and associated disability using a headache diary (documenting headache days on a calendar downloaded using a REDCap link) in the month before initiating *CC* and 2 months after the conclusion of the intervention (to evaluate intermediate effects). Headache-related burden was noted daily on this diary using a colored coded model, the Traffic Light Headache Diary^26^ whereby participants described the maximum disability level of the day using three colors: (1) GREEN indicated a mild headache and/or associated symptoms (light, sound intolerance, nausea, cognitive fog, etc.) without significant disability, meaning the participant could “go” about their day; (2) YELLOW indicated a headache and/or associated symptoms, which reached moderate severity or severe enough to interfere with functioning and caused them to “slow down” activities; and (3) RED indicated a severe, disabling headache and/or associated symptoms, requiring them to fully “stop” their activities. A Traffic Light score was obtained by assigning 3 points for a RED day, 2 points for a YELLOW day and 1 point for a GREEN day. Days with no headache or headache-related symptoms received 0 points for a “Crystal Clear Day.” Participants were contacted by the research team up to three times throughout the program to encourage them to complete headache diaries.

Finally, several outcomes of psychosocial well-being and disability were collected at baseline, after the completion of the program and 2 months after the program. Validated self-report questionnaires included the Headache Impact Test-6 (HIT-6)^27^ to measure headache-related disability, the Patient Health Questionnaire-8 (PHQ-8)^28^ to measure depression, PROMIS Anxiety Short-Form 8a^29^ to measure anxiety, 20-item post-traumatic stress disorder checklist for DSM-5 (PCL-5)^30^ to measure trauma symptoms and the 8-item PROMIS Global Health^31^ to measure function. Five potential mechanisms of change were assessed using validated questionnaires: Emotional Approach Coping Scales-8 (EACS-8)^32^ to measure emotional coping; 10-item Cognitive and Affective Mindfulness Scale – Revised (CAMS-R)^33^ to measure mindfulness; 13-item Pain Catastrophizing Scale (PCS)^34^ to measure catastrophizing; Survey of Pain Attitudes Emotion subscale^35^ to measure pain attitude; and 12-item Self-Compassion Scale – Short Form^36^ to measure self-compassion.

### Statistical analyses

Descriptive statistics were calculated for baseline demographics, characteristics of the group and STTS-R satisfaction measures, with means and standard deviations for continuous measures and numbers and percentages for categorical measures. For exploratory purposes only, we compared the mean headache frequency (days per month) and mean headache burden (Traffic Light score) before and 2 months after the intervention using paired *t*-tests.^37^ For any given day, the worst of the three levels of headache burden was taken as the score using the points system described earlier. For all psychosocial scales, we compared the scores post-treatment vs. baseline and at 2-month follow-up vs. baseline using paired *t*-tests. All analyses were carried out in STATA Version 18.1. As this was a pilot study, we set *p* < 0.05 as the threshold for statistical significance for all tests as opposed to applying a Bonferroni or other correction for multiple comparisons, as one would do in a randomized controlled trial. It is commonplace to use relaxed *p*-values to aid in the interpretation of results in pilot studies.^38^

## Results

### Participant characteristics

Participant characteristics are listed in Table 2. Most participants were women (88%) and female (94%) with post-secondary education (85%) and Caucasian (85%). The mean age of participants was 44.7 years (SD 12.0), and the mean age at headache onset was 19.7 years (SD 9.5). Most participants had chronic migraine (73%), taking acute (94%) and preventive (85%) medications. Almost all had at least one chronic pain condition comorbidity (88%), and 79% had comorbid psychiatric conditions. A trauma history was found in 94% of participants, and 72% had a history of ACEs.

**Table 2.** Baseline demographics, clinical characteristics, retention and satisfaction

	Overall (*n* = 33)
Age, mean (SD)	44.7 (12.0)
Age of headache onset, mean (SD)	19.7 (9.5)
Gender, woman, *n* (%)	29 (87.9%)
Sex, female, *n* (%)	31 (93.9%)
Post-secondary education, *n* (%)	28 (84.8%)
Married or living with partner, *n* (%)	21 (63.6%)
Working, full/part-time, *n* (%)	20 (60.6%)
Household income (*n* = 30), *n* (%) <$60,000 $60,000–$100,000 >$100,000	8 (24.2%) 8 (24.2%) 12 (36.4%)
Race/ethnicity, *n* (%) White Asian Other	28 (84.8%) 3 (9.1%) 6 (18.2%)
Baseline headache diagnosis, *n* (%) Chronic migraine High-frequency episodic headache	24 (72.7%) 7 (21.2%)
Headache days/month, n (SD)	21.6 (8.7)
Headache medications, *n* (%) Acute Preventative	31 (93.9%) 28 (84.8%)
# of pain comorbidities (*n* = 33), *n* (%) 0 1–2 3+	4 (12.1%) 17 (51.5%) 12 (36.4%)
Psychiatric comorbidity, *n* (%)	26 (78.8%)
Trauma history (LEC-5), experienced or witnessed, *n* (%)	31 (93.9%)
Adverse childhood experiences, *n* (%)	24 (72.3%)
STTS-R (/60), mean (SD)	47.6 (10.4)
# of sessions attended (/9), mean (SD)	7.8 (1.5)

LEC-5 = Life Events Checklist for DSM-5; STTS-R = Satisfaction with Therapy and Therapist Scale-Revised.

### Feasibility and acceptability

**Figure 2.**
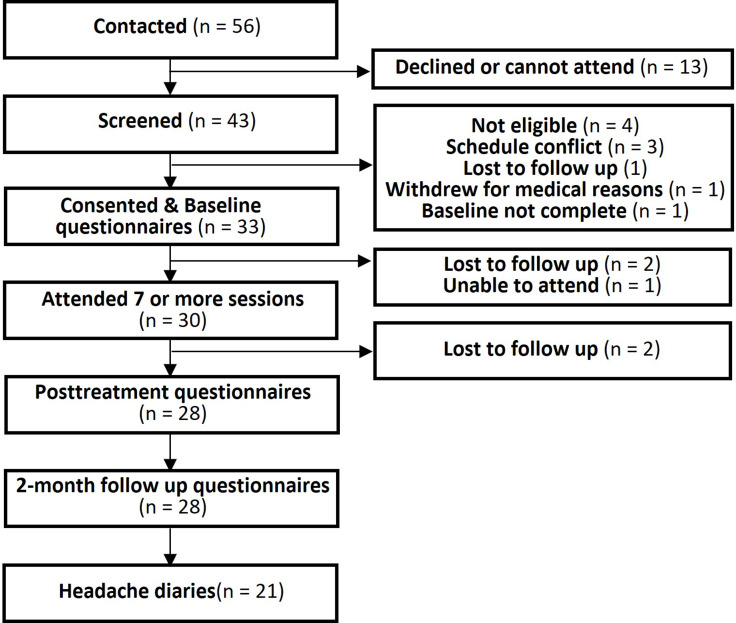
Participant flow through the study.

The feasibility outcome criteria were met. All group cycles and data collection were completed within 13 months, and 33 participants were enrolled, with 10–12 in each group, meeting recruitment targets. Figure 2 depicts participant recruitment and flow through the study. Out of 56 people initially contacted, 33 consented and completed baseline questionnaires, while 16 could not attend or declined, and 4 were found not eligible. Thirty (91%) of 33 participants completed at least 7 of 9 sessions, surpassing the standard 80% threshold. The mean number of sessions attended by each participant was 7.76 (SD 1.5). Two participants were lost to follow-up, and one could no longer attend. Out of the 33 enrolled participants, 28 (85%) completed post-treatment questionnaires, and the same number completed follow-up questionnaires, while 21 (64%) completed headache diaries the month before and 2 months after the intervention. One participant completed post-treatment questionnaires but not the 2-month follow-up questionnaire, and another participant completed the 2-month follow-up but not the immediate post-treatment questionnaire. Therefore, 27 (82%) participants completed all 3 questionnaires. In addition, 23 (70%) completed the HIT-6, and 21 (64%) completed daily headache diaries before and at 2-month post-intervention.

After the program, the mean STTS-R satisfaction score was 47.6 (SD 10.4). On the global satisfaction subscale, 24 out of 28 (86%) participants felt the program led to improvements (i.e., scores ≥ 4 out of 5 points). No participants demonstrated persistent deterioration over the course of the trial.

### Changes in outcomes and mechanisms

**Figure 3.**
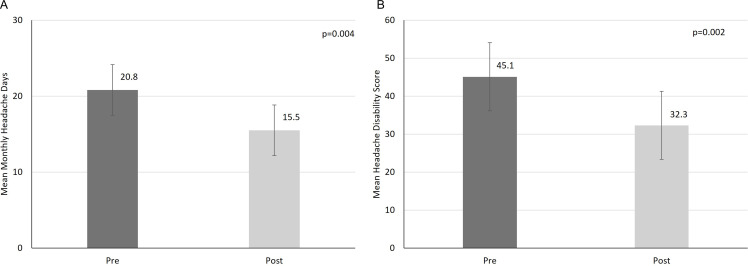
Headache frequency and disability score before and 2 months after Creating Calm. *Note:* Mean headache days per month (A), reduced from 20.8 (95% Cl 17.5–24.1) at baseline (pre) to 15.5 (95% Cl 12.2–18.8) at 2 months after treatment (post). Mean headache disability (B), measured using the Traffic Light Headache score, reduced from 45.1 (95% Cl 36.1–54.1) at baseline (pre) to 32.3 (95% Cl 24.5–40.1) at 2 months after treatment (post).

Headache frequency, headache disability and several measures of psychosocial well-being (Table 3) showed a signal of improvement defined as uncorrected *p* < 0.05. Mean headache days per month reduced from 20.8 (SD 7.6) at baseline to 15.5 (SD 7.8) 2 months after the intervention (*p* = 0.004) (Figure 3). Mean headache disability measured using the Traffic Light score reduced from 45.1 (SD 20.4) at baseline to 32.3 (SD 18.3) 2 months after the intervention (*p* = 0.002) (Figure 3). Changes in measures of headache frequency, disability, functioning and mental health symptoms at baseline, post-treatment and at 2-month follow-up are listed in Table 3, and potential mechanisms of change are listed in Table 4. Improvements were noted in global mental health immediately after the intervention and at the 2-month follow-up period and in headache disability (HIT-6) and depression (PHQ-8) scores at the 2-month follow-up period (Table 3). Potential mechanisms of mindfulness, pain attitudes and self-compassion also showed a signal of improvement immediately after the intervention and at the 2-month follow-up period, while pain catastrophizing (PCS) and emotional coping (EACS-8) showed a signal of improvement at the 2-month follow-up period (Table 4).

**Table 3.** Pilot outcome comparison at baseline, post and 2 months after *Creating Calm* (*n* = 27)

Outcome (measure, range)	Mean (SD)			*t (df)*		*p*	
	T1	T2	T3	T1–T2	T1–T3	T1–T2	T1–T3
Headache disability** (HIT-6, 0–78)	64.1 (3.3)		61.6 (6.0)		2.2 (−2.5)		0.038*
Global physical health (PROMIS GH, 4–20)	12.6 (1.9)	13.2 (1.9)	13.1 (2.3)	−1.6 (−0.6)	−1.4 (−0.4)	0.13	0.18
Global mental health (PROMIS GH, 4–20)	9.5 (2.1)	10.7 (2.6)	11.0 (3.0)	−3.8 (−1.2)	−2.8 (−1.5)	0.0009*	0.0095*
Depression (PHQ-8, 0–24)	9.4 (4.8)	8.2 (5.3)	7.9 (5.6)	2.1 (1.3)	2.1 (1.6)	0.050	0.049*
Trauma symptoms (PCL-5, 0–80)	24.9 (12.4)	23 (12.1)	22.6 (12.9)	0.8 (1.9)	0.91 (2.3)	0.49	0.37
Anxiety (PROMIS A SF8a, 8–40)	22.4 (6.0)	22.6 (5.4)	20.4 (6.4)	0.8 (0.9)	1.6 (2.0)	0.42	0.13

* *p* < 0.05.

** Headache disability, *n* = 23. T1 = baseline data, T2 = post-treatment, T3 = 2-month follow-up.

HIT-6 = Headache Impact Test-6; PROMIS GH = PROMIS Global Health; PHQ-8 = Patient Health Questionnaire-8; PCL-5 = post-traumatic stress disorder checklist for DSM-5; PROMIS A SF8a = PROMIS Anxiety Short-Form 8a.

**Table 4.** Potential mechanisms of change at baseline, post and 2 months after *Creating Calm* (*n* = 27)

Potential mechanism (measure, range)	Mean (SD)			*t (df)*		*p*	
	T1	T2	T3	T1–T2	T1–T3	T1–T2	T1–T3
Emotional coping (EACS-8, 0–32)	18.9 (4.9)	20.1 (4.5)	20.7 (4.0)	−1.5 (−1.3)	−2.3 (−1.8)	0.15	0.033*
Mindfulness (CAMS-R, 10–40)	21.4 (4.5)	23.4 (4.2)	23.7 (4.5)	−2.6 (−2.0)	−2.9 (−2.3)	0.014*	0.0071*
Pain attitudes (SOPA-E, 0–28)	16.9 (3.9)	18 (4.6)	18.3 (5.1)	−2.1 (−1.1)	−2.4 (−1.4)	0.047*	0.022*
Pain catastrophizing (PCS, 0–52)	20.6 (11.4)	18 (10.2)	16.2 (9.6)	1.72 (2.6)	2.7 (4.4)	0.11	0.012*
Self-compassion (SCS-SF, 1–5)	2.6 (0.5)	2.8 (0.6)	2.9 (0.6)	−2.8 (−0.3)	−3.3 (−0.3)	0.01*	0.0030*

* *p* < 0.05. T1 = baseline data, T2 = post-treatment, T3 = 2-month follow-up.

EACS-8 = Emotional Approach Coping Scale-8; CAMS-R = Cognitive and Affective Mindfulness Scale – Revised; SOPA-E = Survey of Pain Attitudes Emotion subscale; PCS = Pain Catastrophizing Scale; SCS-SF = Self-Compassion Scale – Short Form.

## Discussion

This open-label prospective pragmatic real-world pilot study showed that *CC*, a psychological group-based telehealth intervention, is feasible and acceptable for high-frequency episodic and chronic headache in patients with significant comorbidity and adversity history who are managed at a tertiary care clinic. The sample in the current study had substantial pain and psychiatric comorbidity, trauma and childhood adversity prevalence that was much higher than the migraine population in general,^39^ which may be explained by the treatment-refractory population of headache and migraine patients that often present to such a tertiary care setting.^3^ This intervention, used in a complex population that is often excluded from clinical trials, considers the need for equity and accessibility.

Our intervention was well-received and met feasibility thresholds. The attrition rate in the current study was much lower than expected (9%) and compared favorably to a 26% dropout rate from a meta-analysis of CBT groups.^24^ Survey completion was also generally good (85%) despite the complex patient population. Satisfaction was comparable to previous trials for EAET in chronic pain, despite the complex nature of our participant population with chronic migraine and multiple comorbidities in a tertiary headache clinic.^9,10,12^

Results also suggest that *CC*, along with standard care, may merit further testing in a randomized controlled trial for reducing headache frequency (mean reduction of 5.3 headache days per month from pretreatment to post-treatment, representing a 25% reduction). However, since this is a pilot exploratory feasibility study with a small sample size and an uncontrolled design, we cannot conclude efficacy. The current study also suggests a reduction in headache burden using the Traffic Light score. In addition, global mental health showed a signal of improvement immediately after treatment, and this effect was sustained 2 months later, along with headache disability and depression. This intervention’s effect is hypothesized to be mediated by improvements in emotional coping, pain attitudes, pain catastrophizing, mindfulness and self-compassion. These processes were chosen as potential mediators of change as they are each targeted by components of the conceptual model for *CC* (Figure 1): EAET targeting emotional processing or coping; CBT and EAET reversing cognitive distortions, including those related to pain attitudes and catastrophizing; and MBIs targeting mindfulness and self-compassion that are involved in recognizing cognitive distortions, experiencing emotions and anxiety regulation.

While feasibility and acceptability were demonstrated and findings are encouraging, there were several limitations to this study. The small sample size and lack of a control group are common in pilot studies. We cannot definitively conclude the effects of the intervention on headache outcomes, burden or psychological well-being as these were studied as exploratory outcomes, with a relaxed, non-corrected threshold for interpreting significance.^38^ The low completion rate of headache diaries may increase bias and will be a key focus for adaptation in a future study design. Previous studies have shown that headache diary completion improves significantly when using electronic diaries compared to paper diaries.^40,41^ A future study can focus on increasing the headache diary completion rate by sending the headache diary daily in an electronic format with additional reminders (e.g., phone or text). The completion rate may also be impacted by the clinically complex patient population, with a high psychiatric, pain, trauma and ACEs comorbidity profile. In order to maintain recruitment inclusive of complexity, a lower rate of completion may also need to be considered for future sample size determination. Although anonymous feedback questionnaires were administered to group participants to inform modifications of future group iterations, the current study did not include a person with lived experience to directly inform study design and development. In addition, a future larger-scale study would benefit from tracking adverse events to the program and changes to medication use over the course of the trial, as well as quantifying the number of ACEs and comorbidity profile using validated questionnaires.

## Conclusion

*CC* is a promising intervention demonstrating a high level of adherence and satisfaction, as well as encouraging outcomes in a population with high headache burden (mostly chronic migraine with over 21 years of mean headache duration) and a complex comorbidity profile recruited from a real-world, highly refractory population. However, further research is required. The results of this pilot study suggest that it is feasible to conduct a larger clinical randomized controlled trial to evaluate the efficacy of *CC* on headache frequency to optimize non-pharmacological options for those with high-frequency episodic and chronic headache conditions.
